# Passive thigh heating increases peak torque but does not attenuate declines in force production during repeated isokinetic knee extensor exercise in older adults

**DOI:** 10.14814/phy2.71105

**Published:** 2026-09-21

**Authors:** Desmond Denny, Daniel C. Low, Oliver R. Gibson

**Affiliations:** ^1^ Department of Sport, Health and Exercise Sciences Brunel University of London Uxbridge UK; ^2^ Centre for Physical Activity in Health and Disease (CPAHD) Brunel University of London Uxbridge UK

**Keywords:** force development, hyperthermia, muscle strength, older adults, passive heating, thermal therapy, torque

## Abstract

Aging is associated with declines in muscle function, leading to increased functional fatigability. Passive heating has demonstrated positive effects on muscle function during brief exercise in older adults, but the impact of heating on repeated contractions remains unknown. This study examined the effects of passive thigh heating on torque production during repeated and prolonged maximal dynamic isokinetic exercise. Fifteen healthy older adults (68 ± 8 years) completed a protocol involving 90 min of unilateral thigh heating (50°C) via a custom garment (HEAT), while the contralateral limb served as an unheated control (CONT). Participants performed 30 maximal isokinetic knee extensions at 180°/s before and after heating to assess peak torque during a single contraction, and average torque and total work done. Peak torque increased by 6 ± 7 N.m in HEAT (from 74 ± 33 N.m to 81 ± 33 N.m, +9%, *p* < 0.05), whereas CONT saw a no significant change (73 ± 31 N.m to 76 ± 28 N.m). Average torque declined significantly (*p* < 0.05), with decreases from the first third (68 ± 30 N.m) to the middle (57 ± 26 N.m) and final third of the fatigue task (51 ± 22 N.m). There was no difference in averaged torque thirds between HEAT and CONT highlighting that fatigue resistance was unaltered by the intervention. Total work done showed a non‐significant 7% increase in HEAT (from 1619 ± 1100 J to 1751 ± 976 J, *p* = 0.273), a non‐significant decrease of −4% was noted in CONT (from 1589 ± 790 J to 1528 ± 784 J *p* = 0.284). Passive heating enhanced peak torque production, but not fatigue resistance or work done during repetitive dynamic contractions in older adults indicating that the acute benefits of passive heating, may not extend to sustained or repetitive tasks.

## 1 INTRODUCTION

Muscular fatigue is often defined as an exercise‐induced decline in the ability of the muscle to generate force or power (Enoka & Duchateau, 2008). Fatigue can be quantified in a research setting as the reduction in total work done and/or a reduction in torque produced during a continuous or repeated maximal exercise task. Muscular fatigue can also be observed through a reduction in athletic performance (Enoka & Duchateau, 2008), and the inability to sustain the force required to perform daily living tasks (Ma et al., 2011).

Exercise induced muscular fatigue has been observed in a variety of contraction types (Gomes et al., 2021; Kroll, 1973; Westerblad et al., 1998) and is a complex phenomenon, which is influenced by multiple factors that may be peripheral or central in origin (Enoka & Duchateau, 2008). Peripheral fatigue results from changes within the muscle itself, including metabolic disturbances and impaired excitation‐contraction coupling (Gandevia, 2001). Central fatigue arises from impairments in the central nervous system, such as reduced motor neuron output or affected by the motivation of the individual (Gandevia et al., 1995).

It is well‐established that aging negatively impacts neuromuscular physiology, leading to declines in muscle function (Lexell et al., 1988; Wu et al., 2020), such as susceptibility of a muscle to experience a decline in the ability to generate force over time, particularly during sustained or repetitive activity (Hunter, 2018). It is believed older adults experience reductions in muscular power due to impaired contractile function within the muscle rather than inadequate voluntary activation (Wrucke et al., 2024). Additionally, older adults are likely more susceptible to muscular fatigue due to age‐related factors such as reduced muscle mass, diminished nerve function, and decreased cardiovascular efficiency, leading to impaired muscle performance and endurance (Santanasto et al., 2015). These musculoskeletal changes often result in reduced performance of daily tasks and consequently impaired quality of life (Brach & VanSwearingen, 2002) manifesting as declines in walking endurance, balance, and physical activity in healthy older adults (Senefeld et al., 2017). The aged muscle also demonstrates a greater reliance on type I muscle fibers and a reduced reserve of type II fibers, which may limit rapid force generation and power output during high‐intensity tasks (Miljkovic et al., 2015). Older adults typically exhibit slower muscle contractile properties, reduced calcium handling efficiency, diminished motor unit firing rates, and impaired microvascular function, all of which contribute to reduced muscle power and increased fatigability during dynamic tasks (Avin & Law, 2011; Reid & Fielding, 2012). These age‐related changes suggest both the acute force and power production and the ability to sustain force output are affected by age and that older muscle may be particularly responsive to interventions that acutely enhance contractile speed and local perfusion.

It has long been known that skeletal muscle force production is highly sensitive to temperature changes (Bennett, 1984; Elmubarak & Ranatunga, 1984; Kössler & Küchler, 1987; Ranatunga, 1977, 1982, 1994; Ranatunga et al., 1987; Roots & Ranatunga, 2008) with evidence pointing towards a role of temperature in muscular fatigue (Roots et al., 2009). Skeletal muscle hyperthermia above resting temperatures has been shown to enhance cross‐bridge cycling kinetics, accelerate calcium release and reuptake within the sarcoplasmic reticulum, and increase motor nerve conduction velocity, collectively resulting in improved contractile speed and peak force production (Rodrigues et al., 2022; Sargeant, 1987). These temperature‐dependent mechanisms are particularly relevant during dynamic contractions, where force production relies on rapid excitation–contraction coupling and efficient cross‐bridge cycling. Beyond these intramuscular effects, elevated muscle temperature enhances local blood flow and microvascular perfusion (Koch Esteves et al., 2021), which may attenuate the development of peripheral fatigue by improving oxygen delivery and metabolite clearance, physiological processes that are commonly compromised with aging (Adelnia et al., 2019; Salvatore et al., 2022). Furthermore, enhanced perfusion has the potential to improve oxygen delivery and facilitate the removal of fatigue‐related metabolic by‐products such as inorganic phosphate and hydrogen ions, which are implicated in the reduction in force production during repeated maximal contractions (Kent‐Braun, 2009). As such, passive heating represents a plausible intervention through which fatigue‐related declines in force production may be positively altered (McGorm et al., 2018; Rodrigues et al., 2020, 2022). By increasing muscle temperature, acute passive heating interventions may therefore temporarily offset age‐related slowing of contractile kinetics and improve neuromuscular contractile function.

Passive heating of skeletal muscle has emerged as a potential ergogenic aid, with increased peak torque (Denny et al., 2025a, 2025b), maximal power output (Sargeant, 1987), and increased distance within a 6 min walk test (Pellinger et al., 2019), and early‐stage muscle force production (Denny et al., 2025a, 2025b; Mornas et al., 2022; Rodrigues et al., 2021) evidenced to date. However, only one study has investigated the effects of passive increases in muscle temperature and fatigue resistance in younger healthy adults (Ramanauskiene et al., 2008), finding no significant impact on dynamic fatigue resistance. Whilst these data (Ramanauskiene et al., 2008) appear unsupportive of the ergogenic benefits of passive heating to attenuate declines in force production during repeated contractions, it has been known for some time that heating interventions designed to improve local muscle function is problematic when core body temperature also increases given this is known to reduce muscular performance through a reduced central drive (Thomas et al., 2006; Thornley et al., 2003). Therefore, in the study by Ramanauskiene et al. (2008) any peripheral benefits local to the exercising limb may have been masked by negative central perturbations. Localized passive heating is a desirable form of thermal therapy as it offers a means of selectively increasing muscle temperature while minimizing systemic thermal strain and negative effects on central motor drive, providing a more targeted approach to examining temperature‐dependent changes in skeletal muscle (Gibson et al., 2023). Furthermore, older populations are characterized by impairments in skeletal muscle perfusion, excitation–contraction coupling, and contractile efficiency (Adelnia et al., 2019; Delbono, 2011; Power et al., 2013; Salvatore et al., 2022), all of which contribute to an increased susceptibility to peripheral fatigue during repeated contractions. As many of these physiological processes are temperature‐dependent, selectively increasing local muscle temperature may provide a strategy to offset age‐related impairments in contractile function. However, the effects of localized passive muscle heating on fatigue resistance in older adults remain largely unexplored. Given the functional importance of maintaining force output during repeated efforts are important for daily living tasks and fall prevention, understanding whether passive heating can attenuate fatigue‐related declines in force production or total work performed has clear relevance for aging populations. Addressing this gap is necessary to determine whether passive heating represents a viable ergogenic aid for improving repeated high force muscle performance in older adults.

The aim of this study was therefore to investigate the effects of 90 min of passive thigh heating on fatigue resistance and to assess the impact of passive heating on single contraction peak torque. It was hypothesized that the protocol will result in decreases in torque production across 30 repetitions, although passive heating would attenuate the decline in torque production and total work capacity. It was further hypothesized that single contraction peak torque would be increased by the heating intervention. In this study, fatigue resistance was quantified using repeated maximal voluntary contractions of the quadriceps during an isokinetic task, with peak torque recorded for each contraction and average torque calculated across each series of ten repetitions to assess both acute force output and the decline in performance over time. Total work performed was derived from the sum of torque across all repetitions, providing an integrated measure of muscular endurance and fatigue resistance.

## 2 METHODS

### 2.1 Participants

Fifteen older adults (eight female, age 68 ± 8, stature 1.65 ± 0.10 m, body mass 67.7 ± 13.1 kg, BMI 24.8 ± 4.4 kg/m^2^, body fat 27% ± 4%), free of known illness and disease, completed the study. All participants were non‐smokers, with no known history of heat intolerance or neuromuscular disorders, and provided written informed consent prior to taking part. The study was approved by the Brunel University of London Research Ethics Committee (44577‐MHR‐Oct/2023‐47671‐3) and was conducted in accordance with the Declaration of Helsinki. The participants in this study are a subset of the older adults from previously published work recruited from local older adult exercise and research groups (Denny et al., 2025a).

### 2.2 Experimental design

Participants visited the laboratory once at either 9:00 or 13:00 having abstained from heavy exercise (e.g. resistance or interval training, prolonged endurance activity or competitive sport), caffeinated drinks and supplements, and alcohol for 24 h prior to the experimental visit. The right leg (60% of participants' dominant limb) was selected to have the thigh heated for 90 min (HEAT) whilst the contralateral limb served as a control (CONT); for a picture of device implementation see Denny et al., 2025b. The participants wore nylon leggings with the experimental thigh (HEAT) wrapped in a custom garment that circulated water at an outlet temperature of 50°C and a survival blanket for a period of 90 min whilst the contralateral control thigh (CONT) was left uncovered. Physiological and functional measurements were assessed at baseline and at 90 min of heating. This data was collected alongside a parallel study (Denny et al., 2025a), whereby four sets of isokinetic leg extensions were performed every 30 min. The implemented water temperature and duration were utilized based upon pilot and experimental work that had examined safety, efficacy and tolerability in younger and older adults.

### 2.3 Physiological measures

The visit began with an assessment of anthropometric and body composition characteristics. Unshod standing stature was recorded using a stadiometer (SECA model 213, Hamburg, Germany), with mass assessed using electronic scales (SECA model 875, Hamburg, Germany) and body fat percentage via bioelectrical impedance (Bodystat 1500 touch, Isle of Man, UK). A handheld temperature device (Brawn Thermoscan 7, Bussigny, Switzerland) was fully inserted into the right ear canal whereby tympanic temperature (*T*_tymp_) was recorded as a surrogate measure for core body temperature. Heart rate (HR) and mean arterial pressure (MAP) were measured via an automated sphygmomanometer placed over the left brachial artery (Carescape V100 VitalSigns Monitor, Bolton). Superficial muscle temperature (*T*_mu_) was recorded using a muscle temperature probe (RS 103–433 K‐type thermocouple, England). The probe was inserted, without local anesthesia, ~30 mm below the skin surface at a 45° to the horizontal into the vastus lateralis via an 18‐gauge hypodermic needle (Microlance 3, Ireland). *T*_mu_ was manually recorded following temperature stabilization (typically ~5 s) with the probe and guide needle removed thereafter. Wireless iButton (DS1922L Thermochron Data Logger, UK) sensors were placed over the muscle belly of the vastus lateralis and used to measure thigh skin temperature (*T*_skin_) at 60 s intervals. Muscle temperature measurements were an optional measure that all participants were asked if they were happy to complete; only three participants agreed to the measure.

### 2.4 Muscle function assessment

The 30‐repetition isokinetic fatigue test (McLeland et al., 2016) occurred twice using a dynamometer (Biodex Medical Systems, Shirley, NY, USA), once at baseline and then after the 90 min local passive‐heating intervention. Participants completed one set of 30 maximal effort repetitions at 180°/s at baseline for both the heated limb (HEAT) and control limb (CONT), participants were instructed to “kick as hard and as fast as possible for all 30 repetitions”. As this research was only investigating knee extension participants were instructed to passively allow the knee to flex and return to the starting position. All contractions were conducted through a 75°–175° range of motion. At both timepoints HEAT limb was assessed first, followed by the CONT limb. The test occurred 3 min following the isokinetic leg extension tasks in the parallel study (Denny et al., 2025a). Peak torque values were assessed as the single highest recorded value from the 30 repetitions; the average peak torque was also calculated for the first, middle and final thirds of the protocol i.e., across repetitions 1–10, 11–20 and 21–30 respectively. Total work (torque × angular displacement) was assessed as the total work done, measured in joules (J), conducted in the knee extension phase of the kicking exercise across the 30 repetitions. The Biodex system 4 (Biodex Medical Systems, Shirley, NY, USA) software was used to collect data at 100 Hz.

Surface electromyography (EMG) was measured using wireless bipolar EMG sensors (Delsys Trigno, Boston, MA USA) “placed at 2/3 on the line from the anterior *spina iliaca superior* to the lateral side of the patella” in accordance with Seniam recommendations (Stegeman & Hermens, 2007) for the *vastus lateralis*. EMG acquisition software (Delsys Discover, Boston, MA USA) was used to collect the raw EMG data from the EMG sensors. Once exported, the data were processed within a custom MATLAB code. Raw EMG signals were bandpass filtered with a 6–450 Hz cut‐off frequency (Gordon et al. 2023), before subtracting the mean of the signal to correct baseline offsets. The filtered signal then underwent full‐wave rectification and low‐pass filtering to produce a linear envelope using a dual‐pass 2nd order Butterworth filter, after which peak amplitude within each set was reported. EMG signals were standardized by expressing repetitions 2–30 as the percentage decrease relative to repetition 1, enabling comparisons across participants and experimental conditions, these percentage decreases (%Δ) were then grouped into the same repetition blocks (2–10, 11–20 and 21–30) to compare torque production and neural drive activation. EMG data were collected from 10 of the 15 participants. Data collection for the remaining five participants was not possible due to technical malfunction of the EMG equipment. Therefore, the EMG analysis was based on the 10 participants with successfully recorded data only.

### 2.5 Statistical analysis

**FIGURE 1:**
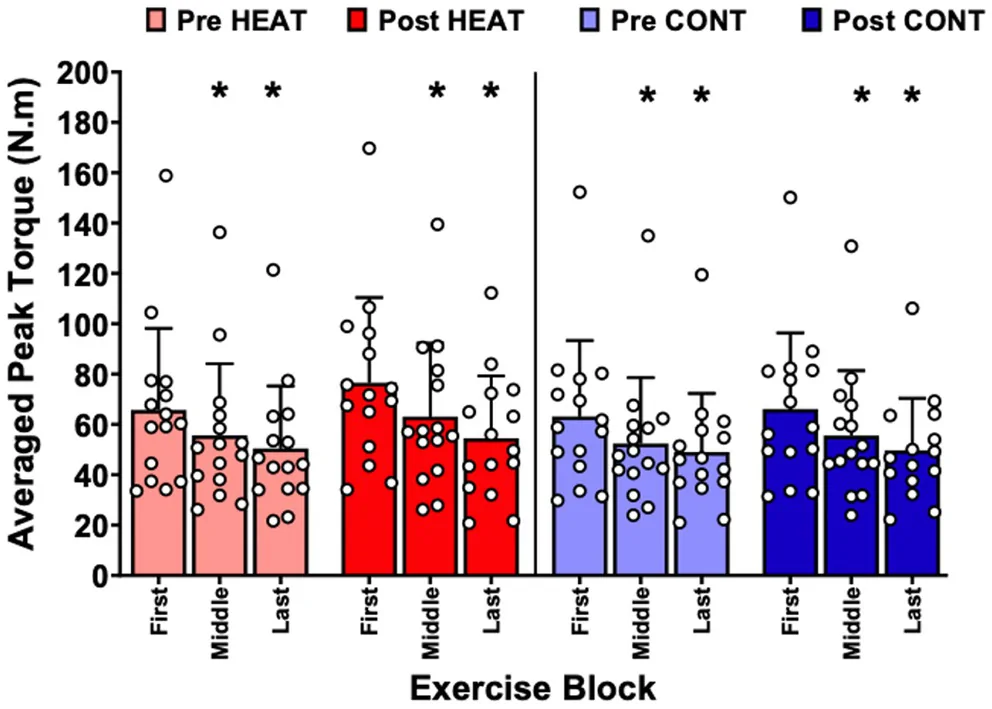
Torque production during the thirty‐repetition isokinetic fatigue task separated into the three ten repetition blocks in the pre HEAT (light red bars), post HEAT (dark red bars), pre CONT (light blue bars) and post CONT (dark blue bars), individual data points are open circles. Data are presented as mean ± SD averaged torque (N.m).

**FIGURE 2:**
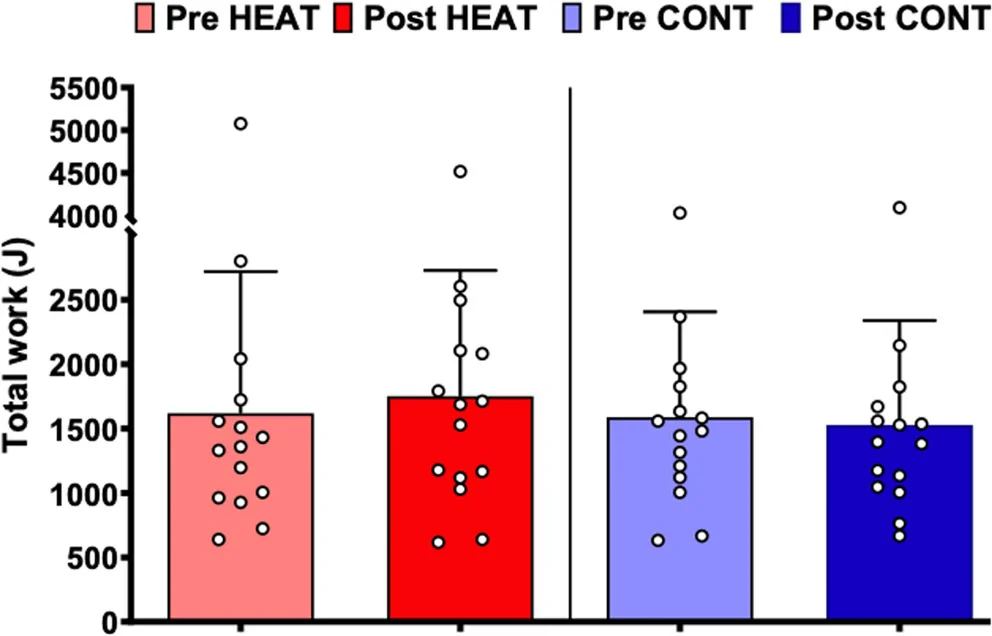
Total work done (J) during the thirty‐repetition isokinetic fatigue task. Bars represent pre HEAT (light red), post HEAT (dark red), pre CONT (light blue) and post CONT (dark blue) assessments with open circles corresponding to individual data points (*n* = 15). Data are presented as mean ± SD.

Statistical significance was set at *p* < 0.05, data are reported Mean ± SD. All data were analyzed using SPSS Statistical Software (Version 25, SPSS, Chicago, IL). Analysis of peak torque and peak EMG used a three‐way repeated‐measures Analysis of Variance (ANOVA) to determine main effect differences across timepoints (Two; 0, 90 min), between conditions (Two; HEAT and CONT) and between repetitions (Three torque blocks; First third, Middle third, Final third). A two‐way ANOVA was used to determine main effect differences across timepoints (Two; 0, 90 min), between conditions (Two; HEAT and CONT) in *T*_skin_ average torque and total work done. A paired sample *t*‐test was used to compare differences between timepoints (Two; 0, 90 min) for *T*_tymp_, heart rate, and MAP. As only three participants volunteered to have *T*_mu_ measured, these data were not subject to statistical analysis. Effect sizes were reported as partial eta squared (ηp^2^), with values of 0.01, 0.06, and 0.14 interpreted as small, medium, and large effects, respectively (Cohen, 2013). Although averaged data were analyzed and are reported in Tables 1 and 2, averaged and individual data points are presented in Figures 1 and 2.

**TABLE 1:** Systemic physiological and muscular functional responses at baseline and following the 90 min passive thigh heating intervention.

	Pre‐intervention	Post‐intervention
T_tymp_ (°C)	36.2 ± 0.4	36.4 ± 0.3
Heart rate (b.min^−1^)	69 ± 12	69 ± 10
MAP (mmHg)	102 ± 10	93 ± 12*
T_skin_ (HEAT) (°C)^	30.5 ± 1.7	39.3 ± 1.2*
T_skin_ (CONT) (°C)	30.6 ± 1.5	33.2 ± 1.1*
T_mu_ (HEAT) (°C)	32.2 ± 0.8	37.2 ± 0.7
T_mu_ (CONT) (°C)	32.2 ± 1.0	33.8 ± 0.6
Peak Torque HEAT (N.m)	79 ± 39	86 ± 37*
Peak Torque CONT (N.m)	73 ± 31	76 ± 28
Average total work HEAT (J)	1619 ± 1064	1751 ± 943
Average total work CONT (J)	1589 ± 790	1528 ± 784

*Note*: Values are Mean ± SD (*N* = 15) except T_mu_ (*n* = 3), * denotes significantly different PRE and POST (*p* < 0.05), ^ denotes between condition difference (*p* < 0.05).

**TABLE 2:** Torque production and surface EMG during a 30‐repetition fatigue task, separated into blocks of ten contractions, at baseline and following 90 mins of passive thigh heating.

	First third contractions	Middle third contractions	Final third contractions
*Pre‐intervention*			
Averaged Torque CONT (N.m)	63 ± 29	52 ± 25	49 ± 22
Averaged Torque HEAT (N.m)	66 ± 31	56 ± 27	50 ± 24
EMG amplitude CONT (%Δ)	−0.05 ± 0.07	−1.29 ± 1.06	−1.98 ± 1.85
EMG amplitude HEAT (%Δ)	−0.78 ± 0.90	−3.32 ± 4.48	−4.27 ± 7.60
*Post‐intervention*			
Averaged Torque CONT (N.m)	66 ± 29	56 ± 25*	50 ± 20*
Averaged Torque HEAT (N.m)	77 ± 33	63 ± 28*	55 ± 24*
EMG amplitude CONT (%Δ)	−0.60 ± 1.32	−3.58 ± 4.96	−8.34 ± 17.81
EMG amplitude HEAT (%Δ)	−1.09 ± 1.67	−1.85% ± 1.72	−1.19 ± 0.25

*Note*: Values are Mean ± SD (*n* = 15) except EMG (*n* = 10), * denotes significantly different PRE and POST (*p* < 0.05).

## 3 RESULTS

### 3.1 Physiological responses

T_tymp_ and HR demonstrated no significant change (*p* > 0.05) from pre‐ to post heating (see Table 1). MAP decreased from baseline by 9 ± 13 mmHg when measured after 90 min of passive heating (*t*_(15)_ = 2.7, *p* = 0.015). *T*_skin_ differed when observing main effects within Condition (*F*_(1,13)_ = 246.8, *p* < 0.001, ηp^2^ = 0.95), Timepoint (*F*_(1,13)_ = 202.2, *p* < 0.001, ηp^2^ = 0.94), Condition*Timepoint (*F*_(1,13)_ = 102.0, *p* < 0.001, ηp^2^ = 0.89). A significant interaction effect demonstrated that there was no difference in *T*_skin_ between conditions at baseline (*p* = 0.724); however, that after 90 min of heating significant differences were observed between conditions (*p* < 0.001) (HEAT = 39.3°C ± 1.3°C, CONT = 33.2°C ± 1.2°C). In HEAT, *T*_mu_ was 32.2°C ± 0.8°C at 0 min, increasing to 37.2°C ± 0.7°C at 90 min. The CONT *T*_mu_ was 32.2°C ± 1.0°C at 0 min, and 33.8°C ± 0.6°C at 90 min. The *T*_mu_ was therefore comparable between CONT and HEAT at baseline, with *T*_mu_ increasing by ~5.0°C in HEAT and by ~1.6°C in CONT; for additional information and a graphical representation of these data please see Denny et al. (2025b).

### 3.2 Peak torque production following thigh passive heating

When observing the effect of heating on peak torque production, a main effect of time was revealed (*F*_(1,14)_ = 13.5, *p* = 0.003, ηp^2^ = 0.49) whereby a + 6 ± 7 N.m improvement was observed at the post intervention timepoint. A significant interaction effect of Timepoint*Condition (*F*_(1,14)_ = 3.1, *p* = 0.008, ηp^2^ = 0.38) revealed a significantly higher (*p* = 0.008) post HEAT measurement (81 ± 33 N.m) when compared to pre‐intervention measurement timepoint (74 ± 33 N.m), whereas CONT saw no increase (*p* = 0.171) from baseline (73 ± 31 N.m) to post intervention (76 ± 28 N.m). At baseline no difference was observed in peak torque production between conditions (*p* = 0.379), however, a significant (*p* = 0.027) 10 N.m increase was observed in HEAT compared to CONT post intervention. No other significant main (e.g. repetition block) or interaction effects were observed.

### 3.3 Averaged torque, total work done and vastus lateralis EMG following upper thigh muscle hyperthermia

A main effect of Timepoint was observed (*F*_(1,14)_ = 8.8, *p* = 0.010, ηp^2^ = 0.39), whereby the post testing averaged 61 N.m as opposed to the pre testing averaged 56 N.m, A main effect was observed (*F*_(2,28)_ = 26.9, *p* < 0.001, ηp^2^ = 0.66) whereby the isokinetic fatigue test elicited expected decreases in averaged peak torque production throughout the thirty isokinetic repetitions. Mean torque within the first third of the fatigue test (68 ± 30 N.m) was significantly higher (*p* < 0.001) than the middle third (57 ± 26 N.m) which was significantly higher (*p* < 0.001) than the final third (51 ± 22). An interaction effect was not observed between Timepoint*Condition*Thirds (*p* = 0.250) (Figure 1). No differences were observed for mean torque or averaged torque between HEAT and CONT.

The effect of heating on average total work done during the fatigue task was not significantly different (*p* = 0.62) between pre (1604 ± 953 J) and post (1640 ± 875 J) intervention timepoints. Furthermore, no significant differences (*p* = 0.27) were observed in total work done in HEAT (pre = 1619 ± 1100 J, post = 1751 ± 976 J; change = +133 ± 449 J (+7%)) vs. CONT (*p* = 0.28) (pre = 1589 ± 817 J, post = 1528 ± 813 J, change = −61 ± 213 J (−4%)); *p* = 12, ηp^2^ = 0.16, (Figure 2).

When observing the peak EMG of the *vastus lateralis* during the fatigue test no significant differences (*p* > 0.05) were observed between the two conditions or timepoints or across blocks of contractions (Table 2). As such, EMG magnitudes were statistically unchanged (first third, −1.99 ± 2.81%Δ; middle third, −3.34 ± 7.35%Δ; final third, −3.38 ± 8.53%Δ; *p* = 0.075). Furthermore, there were no interaction effects.

## 4 DISCUSSION

This study investigated the effects of passive thigh heating on the fatigue resistance of knee extensor muscles during isokinetic exercise in healthy older adults. The protocol employed within this study demonstrated a fatiguing effect on the working muscle, as was hypothesized and observed in similar studies (Gomes et al., 2021; Rawson, 2010). The results indicated that 90 min of passive thigh heating increased the skin temperature of the heated limb to levels congruent with studies demonstrating ergogenic benefits (Denny et al., 2025a, 2025b; Rodrigues et al., 2023), however, fatigue resistance and the attenuation of force decline were not observed. A non‐significant +7% increase in total work done was observed in HEAT whilst a −4% change was observed in CONT following the 90 min protocol. In keeping with previous experimental findings, a significant +8% increase in peak muscle force (torque produced during isokinetic contractions at 180°/s) was observed.

The absence of statistically significant improvements in fatigue resistance following localized passive heating aligns with the findings of Ramanauskiene et al. (2008) where whole‐body heating, which increased superficial muscle temperature to 39.5°C ± 0.2°C, did not affect dynamic fatigue resistance in younger adults, but did result in higher peak force output that was similar in magnitude to that observed in the current study. Since both modalities demonstrated no change in fatigue resistance, this suggests that heating, whether local or whole body, does not impact fatiguing exercise in both younger, or older adults.

Whilst fatigue resistance was unchanged statistically, an increase in peak torque was observed in both the HEAT and CONT condition after 90 min of heating aligns with previous work demonstrating the benefits of passive limb heating on neuromuscular function from our group (Denny et al., 2025a, 2025b), and others (Mornas et al., 2021, 2022; Rodrigues et al., 2022, 2023). Passive heating has been previously observed to benefit maximal 6 min walking performance in clinical populations (O'Connor et al., 2025; Pellinger et al., 2019) the lack of observed improvement in the present study may be task dependent or attributed to passive heating being more effective in clinical populations than in healthy older adults. Clinical groups often present with lower baseline function or specific pathologies, such as peripheral artery disease, that are more directly influenced by the physiological effects of passive heating, particularly the increase in local blood flow. Differences in outcomes may stem from the nature of the task, as walking involves repeated submaximal contractions over a limited range of motion, whereas the present study employed maximal, large‐range isokinetic knee extensions.

Surface EMG values did not change between the HEAT or the CONT group suggesting that this passive heating protocol did not reduce or increase neural drive to the working muscle. This null finding was to be expected as the negative alterations in neural drive have previously been attributed to whole body heating whereby core temperature is increased (Thomas et al., 2006), rather than localized passive heating. Whilst a statistically significant difference was not observed between the thirds, a decreasing trend in peak EMG was observable. Based on the work of others (Hsu et al., 2023) this implies that central fatigue is a contributing factor to the observed reduction in force production as the task progressed; however, passive thigh heating did not modulate this outcome. Whole body heating which results in increases in core temperature has demonstrated decreases in neural drive through decreased peak EMG amplitude during maximal isokinetic and isometric exercise (Racinais, 2013; Thomas et al., 2006). Prior work investigating localized passive heating has not demonstrated any reductions in EMG amplitude similar to that observable in whole body heating protocols; therefore, our data align with that of others (Rodrigues et al., 2021).

Localized heating of skeletal muscle has been speculated to facilitate calcium handling within the muscle fiber and at the sarcoplasmic reticulum (Rodrigues et al., 2022), which may enhance muscle contractile properties (Kobayashi et al., 2005; Rodrigues et al., 2022). However, the lack of observed improvements in fatigue resistance during this experiment suggests that if this mechanism is present following an acute exposure to passive heating, it does not provide benefit to this type of exercise test or was not of a sufficient magnitude to offset the progressive onset of fatigue within this 30 repetition task. Metabolic accumulation of hydrogen ions and inorganic phosphate generated form anaerobic respiration within the muscle fiber have been suggested to be a driver of fatigue within older adults during an isokinetic 30 repetition knee extension task (Kent‐Braun, 2009). It was theorized that an increased local blood flow, as seen in prior passive heating work (Koch Esteves et al., 2021; Pearson et al., 2011), would aid in the removal of metabolic byproducts as seen in low intensity active recovery (Zhang et al., 2025) and improve fatigue resistance. However, the benefits of increased blood flow may occur over a longer time period than during maximal exercise, where blood flow may be occluded during peak contraction. The findings of this study underscore the complexity and nuances of applying passive thigh heating as an ergogenic aid in older adults (Rodrigues et al., 2020, 2022). While prior work has highlighted improvements in acute muscle function such as early force production in young and older adults (Denny et al., 2025a) and peak torque in young cohorts (Denny et al., 2025b), the present study indicates that these benefits may not translate to tasks requiring sustained or repetitive contractions in older adults of <60 s in the way that aerobic endurance activity of ~5–10 min in duration is following limb heating in clinical cohorts (Pellinger et al., 2019). As such, passive heating may more selectively enhance the tolerance of submaximal rather than maximal exercise tasks.

### 4.1 Limitations and future directions

This study had several limitations that may have influenced the outcomes. This current study included additional exercise between the two fatigue testing sets, this altered the muscle temperature (and likely the metabolic state), especially in the CONT limb, which prevents a direct comparison between a truly rested heated limb and a truly rested thermoneutral limb. Additionally, there is a need for consideration of cumulative and contralateral fatigue, this study had multiple fatigue tests within a single session, and the testing order could have provided some contralateral fatigue in the CONT limb as it always was performed after HEAT, although long rest periods were provided it is possible that participants were not fully recovered between exercise sets. Superficial muscle temperature was only conducted on 3 participants and was not ultrasound guided, this makes it impossible to know the depth of temperature measurement, and we were unable to say definitively that the probe was settled precisely within the same muscle tissue location. The scope of the surface EMG analysis within this study was not designed to assesses or differentiate central or peripheral fatigue across all active muscles during knee extensor activity with analysis of frequency and amplitude absent, it was designed to provide a simple view of muscle activation between a heated and thermoneutral control limb. Future work should therefore seek to investigate the mechanisms of fatigue during maximal exercise prior to and following local but not systemic hyperthermia. Future work should also aim to expand insights into the influence of exercise task on ergogenic outcomes arising from passive heating, including a variety of muscular contraction types, especially those that more closely relate to daily living tasks. The lack of synchronicity between the EMG and dynamometry restricted the scope of analysis, additionally the absence of an evoked twitch precludes confirmation of a true maximal reference against which voluntary contractions can be compared. The current study investigated only healthy older adults who all were able to independently commute to the laboratory for testing, those who struggle with independent living were not tested within this study, further research may wish to include older adults who are less physically active or have trouble living unassisted, as they stand to benefit from an ergogenic tool to reach optimum muscle function before conducting daily living tasks. Future research should also expand on the clinical applications in the possibility of passive heating to improve total work done in sustained efforts (Pellinger et al., 2019). A mechanistic study that improves knowledge around known and unknown mechanisms influencing muscle function following heating would add greatly to the field. This might include analysis of blood flow, calcium concentrations or muscle‐tendon mechanics following localized exposure to passive heating.

## 5 CONCLUSION

In conclusion, 90 min of passive thigh heating using a custom garment circulating 50°C water did not significantly improve isokinetic fatigue resistance during repeated maximal isokinetic knee extensor exercise in healthy older adults. Overall, these findings indicate that while passive thigh heating may improve some aspects of acute muscle function, the ergogenic effects of the intervention appear to be task specific and may not extend to high intensity isokinetic fatiguing exercise in healthy older adults. Further research is needed to clarify the mechanisms involved and to replicate this effect.

## AUTHOR CONTRIBUTIONS

**Oliver R. Gibson:** Conceptualization; data curation; formal analysis; investigation; methodology; supervision. **Desmond Denny:** Conceptualization; data curation; formal analysis; methodology; project administration; resources. **Daniel C. Low:** Conceptualization; data curation; formal analysis; investigation; methodology; project administration; supervision.

## FUNDING INFORMATION

No external funding was provided.

## ETHICS STATEMENT

The study was approved by the Brunel University of London Research Ethics committee (44577‐MHR‐Oct/2023‐ 47671‐3). All procedures were conducted following written informed consent, in accordance with the Declaration of Helsinki.

## References

[phy271105-bib-0001] Adelnia, F. , Cameron, D. , Bergeron, C. M. , Fishbein, K. W. , Spencer, R. G. , Reiter, D. A. , & Ferrucci, L. (2019). The role of muscle perfusion in the age‐associated decline of mitochondrial function in healthy individuals. Frontiers in Physiology, 10, 427. 10.3389/fphys.2019.00427 PMC6473080 31031645

[phy271105-bib-0055] Avin, K. G. , & Law, L. A. (2011). Age‐related differences in muscle fatigue vary by contraction type: A meta‐analysis. Physical Therapy, 91(8), 1153–1165. 10.2522/ptj.20100333. Epub 2011 May 26. PMC3145894 21616932

[phy271105-bib-0002] Bennett, A. F. (1984). Thermal dependence of muscle function. American Journal of Physiology. Regulatory, Integrative and Comparative Physiology, 247, R217–R229. 10.1152/ajpregu.1984.247.2.r217 6380314

[phy271105-bib-0003] Brach, J. S. , & VanSwearingen, J. M. (2002). Physical impairment and disability: Relationship to performance of activities of daily living in community‐dwelling older men. Physical Therapy, 82, 752–761. 12147005

[phy271105-bib-0058] Cohen, J. (2013). Statistical power analysis for the behavioral sciences (2nd ed.). Routledge.

[phy271105-bib-0004] Delbono, O. (2011). Expression and regulation of excitation‐contraction coupling proteins in aging skeletal muscle. Current Aging Science, 4, 248–259. 10.2174/1874609811104030248 PMC9634721 21529320

[phy271105-bib-0005] Denny, D. , Low, D. C. , & Gibson, O. R. (2025a). Passive thigh heating improves peak force production in younger adults and early isokinetic force production in younger and older adults. Experimental Physiology. 10.1113/EP092690. Epub ahead of print. PMC13394178 41214854

[phy271105-bib-0006] Denny, D. , Low, D. C. , & Gibson, O. R. (2025b). Passive thigh heating improves isokinetic but not isotonic muscle function. Journal of Thermal Biology, 130, 104156. 10.1016/j.jtherbio.2025.104156 40483773

[phy271105-bib-0007] Elmubarak, M. H. , & Ranatunga, K. W. (1984). Temperature sensitivity of tension development in a fast‐twitch muscle of the rat. Muscle & Nerve, 7, 298–303. 10.1002/mus.880070408 6727914

[phy271105-bib-0008] Enoka, R. M. , & Duchateau, J. (2008). Muscle fatigue: What, why and how it influences muscle function. The Journal of Physiology, 586, 11–23. 10.1113/jphysiol.2007.139477 PMC2375565 17702815

[phy271105-bib-0009] Gandevia, S. C. (2001). Spinal and supraspinal factors in human muscle fatigue. Physiological Reviews, 81, 1725–1789. 10.1152/physrev.2001.81.4.1725 11581501

[phy271105-bib-0010] Gandevia, S. C. , Allen, G. M. , & McKenzie, D. K. (1995). Central fatigue. Advances in Experimental Medicine and Biology, 384, 281–294. 8585457

[phy271105-bib-0011] Gibson, O. R. , Astin, R. , Puthucheary, Z. , Yadav, S. , Preston, S. , Gavins, F. N. E. , & González‐Alonso, J. (2023). Skeletal muscle angiogenic, regulatory, and heat shock protein responses to prolonged passive hyperthermia of the human lower limb. American Journal of Physiology. Regulatory, Integrative and Comparative Physiology, 324, R1–R14. 10.1152/ajpregu.00320.2021 36409025

[phy271105-bib-0012] Gomes, M. , Santos, P. , Correia, P. , Pezarat‐Correia, P. , & Mendonca, G. V. (2021). Sex differences in muscle fatigue following isokinetic muscle contractions. Scientific Reports, 11, 1–12. 10.1038/s41598-021-87443-0 PMC8046769 33854136

[phy271105-bib-0057] Gordon, R. J. F. H. , Moss, J. N. , Castelli, F. , Reeve, T. , Diss, C. E. , Tyler, C. J. , & Tillin, N. A. (2023). Heat acclimation reduces the effects of whole‐body hyperthermia on knee‐extensor relaxation rate, but does not affect voluntary torque production. European Journal of Applied Physiology, 123(5), 1067–1080. 10.1007/s00421-022-05127-7. Epub 2023 Jan 13. PMC10119217 36637508

[phy271105-bib-0013] Hsu, L. I. , Lim, K. W. , Lai, Y. H. , Chen, C. S. , & Chou, L. W. (2023). Effects of muscle fatigue and recovery on the neuromuscular network after an intermittent handgrip fatigue task: Spectral analysis of electroencephalography and electromyography signals. Sensors, 23, 2440. 10.3390/s23052440 PMC10007140 36904641

[phy271105-bib-0014] Hunter, S. K. (2018). Performance fatigability: Mechanisms and task specificity. Cold Spring Harbor Perspectives in Medicine, 8, a029728. 10.1101/cshperspect.a029728 PMC6027928 28507192

[phy271105-bib-0015] Kent‐Braun, J. A. (2009). Skeletal muscle fatigue in old age: Whose advantage? Exercise and Sport Sciences Reviews, 37, 3–9. 10.1097/JES.0b013e318190ea2e PMC2697566 19098518

[phy271105-bib-0016] Kobayashi, T. , Goto, K. , Kojima, A. , Akema, T. , Uehara, K. , Aoki, H. , Sugiura, T. , Ohira, Y. , & Yoshioka, T. (2005). Possible role of calcineurin in heating‐related increase of rat muscle mass. Biochemical and Biophysical Research Communications, 331, 1301–1309. 10.1016/j.bbrc.2005.04.096 15883017

[phy271105-bib-0017] Koch Esteves, N. , Gibson, O. R. , Khir, A. W. , & González‐Alonso, J. (2021). Regional thermal hyperemia in the human leg: Evidence of the importance of thermosensitive mechanisms in the control of the peripheral circulation. Physiological Reports, 9, e14953. 10.14814/phy2.14953 PMC8339537 34350727

[phy271105-bib-0018] Kössler, F. , & Küchler, G. (1987). Contractile properties of fast and slow twitch muscles of the rat at temperatures between 6 and 42 degrees C. Biomedica Biochimica Acta, 46, 815–822. 3446207

[phy271105-bib-0019] Kroll, W. (1973). Effects of local muscular fatigue due to isotonic and isometric exercise upon fractionated reaction time components. Journal of Motor Behavior, 5, 81–93. 10.1080/00222895.1973.10734953 23952663

[phy271105-bib-0020] Lexell, J. , Taylor, C. C. , & Sjöström, M. (1988). What is the cause of the ageing atrophy?: Total number, size and proportion of different fiber types studied in whole vastus lateralis muscle from 15‐ to 83‐year‐old men. Journal of the Neurological Sciences, 84, 275–294. 10.1016/0022-510x(88)90132-3 3379447

[phy271105-bib-0021] Ma, L. , Chablat, D. , Bennis, F. , Zhang, W. , Hu, B. , & Guillaume, F. (2011). A novel approach for determining fatigue resistances of different muscle groups in static cases. International Journal of Industrial Ergonomics, 41, 10–18.

[phy271105-bib-0022] McGorm, H. , Roberts, L. A. , Coombes, J. S. , & Peake, J. M. (2018). Turning up the heat: An evaluation of the evidence for heating to promote exercise recovery, muscle rehabilitation and adaptation. Sports Medicine, 48, 1311–1328. 10.1007/s40279-018-0876-6 29470824

[phy271105-bib-0023] McLeland, K. A. , Ruas, C. V. , Arevalo, J. A. , Bagley, J. R. , Ciccone, A. B. , Brown, L. E. , Coburn, J. W. , Galpin, A. J. , & Malyszek, K. K. (2016). Comparison of knee extension concentric fatigue between repetition ranges. Isokinetics and Exercise Science, 24, 33–38.

[phy271105-bib-0054] Miljkovic, N. , Lim, J. Y. , Miljkovic, I. , & Frontera, W. R. (2015). Aging of skeletal muscle fibers. Annals of Rehabilitation Medicine, 39(2), 155–162. 10.5535/arm.2015.39.2.155. Epub 2015 Apr 24. PMC4414960 25932410

[phy271105-bib-0024] Mornas, A. , Racinais, S. , Brocherie, F. , Alhammoud, M. , Hager, R. , Desmedt, Y. , & Guilhem, G. (2021). Hyperthermia reduces electromechanical delay via accelerated electrochemical processes. Journal of Applied Physiology, 130, 290–297. 10.1152/japplphysiol.00538.2020 33180642

[phy271105-bib-0025] Mornas, A. , Racinais, S. , Brocherie, F. , Alhammoud, M. , Hager, R. , Desmedt, Y. , & Guilhem, G. (2022). Faster early rate of force development in warmer muscle: An in vivo exploration of fascicle dynamics and muscle‐tendon mechanical properties. American Journal of Physiology. Regulatory, Integrative and Comparative Physiology, 323, R123–R132. 10.1152/ajpregu.00280.2021 35579335

[phy271105-bib-0026] O'Connor, F. K. , Sabapathy, S. , Sharma, P. , Roberts, L. , Walsh, J. R. , Bach, A. J. E. , Hopman, A. G. M. , Louis, M. , Balmain, B. N. , & Morris, N. R. (2025). Acute lower‐limb heating improves exercise performance in individuals with heart failure with reduced ejection fraction. European Journal of Heart Failure, 27, 2168–2169. 10.1002/ejhf.3638 PMC12765035 40044594

[phy271105-bib-0027] Pearson, J. , Low, D. A. , Stöhr, E. , Kalsi, K. , Ali, L. , Barker, H. , & González‐Alonso, J. (2011). Hemodynamic responses to heat stress in the resting and exercising human leg: Insight into the effect of temperature on skeletal muscle blood flow. American Journal of Physiology. Regulatory, Integrative and Comparative Physiology, 300, R663–R673. 10.1152/ajpregu.00662.2010 PMC3064274 21178127

[phy271105-bib-0028] Pellinger, T. K. , Neighbors, C. B. , & Simmons, G. H. (2019). Acute lower leg heating increases exercise capacity in patients with peripheral artery disease. The Journal of Cardiovascular Nursing, 34, 130–133. 10.1097/JCN.0000000000000510 30024488

[phy271105-bib-0029] Power, G. A. , Dalton, B. H. , & Rice, C. L. (2013). Human neuromuscular structure and function in old age: A brief review. Journal of Sport and Health Science, 2, 215–226. 10.1016/j.jshs.2013.07.001 PMC4801513 27011872

[phy271105-bib-0030] Racinais, S. (2013). Hot ambient conditions shift the force/EMG relationship. Springerplus, 2, 1–7. 10.1186/2193-1801-2-317 PMC3715679 23888283

[phy271105-bib-0031] Ramanauskiene, I. , Skurvydas, A. , Sipavičiene, S. , Senikiene, Ž. , Linonis, V. , Krutulyte, G. , & Vizbaraite, D. (2008). Influence of heating and cooling on muscle fatigue and recovery. Medicina, 44, 687. 18971606

[phy271105-bib-0032] Ranatunga, K. W. (1977). Influence of temperature on the characteristics of summation of isometric mechanical responses of mammalian skeletal muscle. Experimental Neurology, 54, 513–532. 10.1016/0014-4886(77)90254-0 844523

[phy271105-bib-0033] Ranatunga, K. W. (1982). Temperature‐dependence of shortening velocity and rate of isometric tension development in rat skeletal muscle. The Journal of Physiology, 329, 465–483. 10.1113/jphysiol.1982.sp014314 PMC1224791 7143257

[phy271105-bib-0034] Ranatunga, K. W. (1994). Thermal stress and Ca‐independent contractile activation in mammalian skeletal muscle fibers at high temperatures. Biophysical Journal, 66, 1531–1541. 10.1016/S0006-3495(94)80944-0 PMC1275873 8061202

[phy271105-bib-0035] Ranatunga, K. W. , Sharpe, B. , & Turnbull, B. (1987). Contractions of a human skeletal muscle at different temperatures. The Journal of Physiology, 390, 383–395. 10.1113/jphysiol.1987.sp016707 PMC1192187 3443940

[phy271105-bib-0036] Rawson, E. S. (2010). Enhanced fatigue resistance in older adults during repeated sets of intermittent contractions. Journal of Strength and Conditioning Research, 24, 251–256. 10.1519/JSC.0b013e3181a8f7cf 19661832

[phy271105-bib-0056] Reid, K. F. , & Fielding, R. A. (2012). Skeletal muscle power: A critical determinant of physical functioning in older adults. Exercise and Sport Sciences Reviews, 40(1), 4–12. 10.1097/JES.0b013e31823b5f13 PMC3245773 22016147

[phy271105-bib-0037] Rodrigues, P. , Orssatto, L. B. R. , Trajano, G. S. , Wharton, L. , & Minett, G. M. (2023). Increases in muscle temperature by hot water improve muscle contractile function and reduce motor unit discharge rates. Scandinavian Journal of Medicine & Science in Sports, 33, 754–765. 10.1111/sms.14312 36610040

[phy271105-bib-0038] Rodrigues, P. , Trajano, G. S. , Stewart, I. B. , & Minett, G. M. (2022). Potential role of passively increased muscle temperature on contractile function. European Journal of Applied Physiology, 122, 2153–2162. 10.1007/s00421-022-04991-7 PMC9463203 35771296

[phy271105-bib-0039] Rodrigues, P. , Trajano, G. S. , Wharton, L. , & Minett, G. M. (2020). Effects of passive heating intervention on muscle hypertrophy and neuromuscular function: A preliminary systematic review with meta‐analysis. Journal of Thermal Biology, 93, 102684. 10.1016/j.jtherbio.2020.102684 33077110

[phy271105-bib-0040] Rodrigues, P. , Trajano, G. S. , Wharton, L. , Orssatto, L. B. R. , & Minett, G. M. (2021). A passive increase in muscle temperature enhances rapid force production and neuromuscular function in healthy adults. Journal of Science and Medicine in Sport, 24, 818–823. 10.1016/j.jsams.2021.01.003 33487572

[phy271105-bib-0041] Roots, H. , Ball, G. , Talbot‐Ponsonby, J. , King, M. , McBeath, K. , & Ranatunga, K. W. (2009). Muscle fatigue examined at different temperatures in experiments on intact mammalian (rat) muscle fibers. Journal of Applied Physiology (Bethesda, MD: 1985), 106, 378–384. 10.1152/japplphysiol908832008 PMC2644245 19057001

[phy271105-bib-0042] Roots, H. , & Ranatunga, K. W. (2008). An analysis of the temperature dependence of force, during steady shortening at different velocities, in (mammalian) fast muscle fibres. Journal of Muscle Research and Cell Motility, 29, 9–24. 10.1007/s10974-008-9138-9 PMC2493522 18523851

[phy271105-bib-0043] Salvatore, S. S. , Zelenski, K. N. , & Perkins, R. K. (2022). Age‐related changes in skeletal muscle oxygen utilization. Journal of Functional Morphology and Kinesiology, 7(4), 87. 10.3390/jfmk7040087 PMC9590092 36278748

[phy271105-bib-0044] Santanasto, A. J. , Glynn, N. W. , Jubrias, S. A. , Conley, K. E. , Boudreau, R. M. , Amati, F. , Mackey, D. C. , Simonsick, E. M. , Strotmeyer, E. S. , Coen, P. M. , Goodpaster, B. H. , & Newman, A. B. (2015). Skeletal muscle mitochondrial function and fatigability in older adults. The Journals of Gerontology Series A: Biological Sciences and Medical Sciences, 70, 1379–1385. 10.1093/gerona/glu134 PMC4612379 25167867

[phy271105-bib-0045] Sargeant, A. J. (1987). Effect of muscle temperature on leg extension force and short‐term power output in humans. European Journal of Applied Physiology and Occupational Physiology, 56, 693–698. 10.1007/BF00424812 3678224

[phy271105-bib-0053] Senefeld, J. , Yoon, T. , & Hunter, S. K. (2017). Age differences in dynamic fatigability and variability of arm and leg muscles: Associations with physical function. Experimental Gerontology, 87(Pt A), 74–83. 10.1016/j.exger.2016.10.008. Epub 2016 Oct 27. PMC5362345 27989926

[phy271105-bib-0046] Stegeman, D. F. , & Hermens, H. J. (2007). Standards for surface electromyography: the European project “Surface EMG for non‐invasive assessment of muscles (SENIAM)”.

[phy271105-bib-0047] Thomas, M. M. , Cheung, S. S. , Elder, G. C. , & Sleivert, G. G. (2006). Voluntary muscle activation is impaired by core temperature rather than local muscle temperature. Journal of Applied Physiology, 100, 1361–1369. 10.1152/japplphysiol.00945.2005 16339343

[phy271105-bib-0048] Thornley, L. J. , Maxwell, N. S. , & Cheung, S. S. (2003). Local tissue temperature effects on peak torque and muscular endurance during isometric knee extension. European Journal of Applied Physiology, 90(5), 588–594. 10.1007/s00421-003-0927-y 12923644

[phy271105-bib-0049] Westerblad, H. , Allen, D. G. , Bruton, J. D. , Andrade, F. H. , & Lännergren, J. (1998). Mechanisms underlying the reduction of isometric force in skeletal muscle fatigue. Acta Physiologica Scandinavica, 162, 253–260. 10.1046/j.1365-201X.1998.0301f.x 9578370

[phy271105-bib-0050] Wrucke, D. J. , Kuplic, A. , Adam, M. D. , Hunter, S. K. , & Sundberg, C. W. (2024). Neural and muscular contributions to the age‐related differences in peak power of the knee extensors in men and women. Journal of Applied Physiology (1985), 137, 1021–1040. 10.1152/japplphysiol.00773.2023 PMC11486474 39205638

[phy271105-bib-0051] Wu, R. , de Vito, G. , Delahunt, E. , & Ditroilo, M. (2020). Age‐related changes in motor function (I). Mechanical and neuromuscular factors. International Journal of Sports Medicine, 41, 709–719. 10.1055/a-1144-3408 32365388

[phy271105-bib-0052] Zhang, X. , Zhang, G. , Pang, X. , Li, X. , Yao, Y. , Liu, Y. , Bi, Y. , Sha, M. , & Zhang, X. (2025). Evaluating the impact of self myofascial release and traditional recovery strategies on volleyball athletes using thermal imaging and biochemical assessments. Scientific Reports, 15, 1–17. 10.1038/s41598-025-91193-8 PMC11846910 39987358

